# The relationship between accessibility of urban and rural high schools and enrollment rate: A county-level study

**DOI:** 10.1371/journal.pone.0325263

**Published:** 2025-06-10

**Authors:** Xinlong Li, Sharifah Salwa Syed Mahdzar, Khairul Anwar Mohamed Khaidzir, Iziq Eafifi Ismail

**Affiliations:** 1 Faculty of Built Environment and Surveying, Universiti Teknologi Malaysia, Johor Bahru, Johor, Malaysia; 2 Centre for the Study of Built Environment in the Malay World (KALAM) and Institute for Smart Infrastructure and Innovative Construction (ISIIC), Universiti Teknologi Malaysia; Ministry of Health, Sri Lanka, SRI LANKA

## Abstract

The study investigates the relationship between high school accessibility and enrollment rates in urban and rural areas within a county. The main goal is to explore how spatial accessibility to high schools impacts student enrollment opportunities, particularly in the context of urban-rural disparities. Using empirical analysis, the study evaluates the effects of transportation infrastructure, school distribution, and government policies on educational access and fairness. The findings reveal, there is no significant correlation between high school accessibility and enrollment rates in both urban and rural areas. Furthermore, the research shows that improvements in rural infrastructure and policies have helped mitigate potential disparities, leading to a more balanced educational landscape. This study provides a theoretical framework for assessing high school accessibility and its impact on enrollment rates, offering valuable insights for policy makers looking to optimize educational resources and promote fairness. The findings highlight the need for further research with larger samples and the inclusion of additional variables to understand the broader impact of accessibility on education.

## 1. Introduction

With the increasing focus of the Chinese government on education, achieving the equitable distribution of educational resources, particularly realizing urban-rural educational equity, has become a key concern for both scholars and policymakers. Urban-rural educational equity involves not only the distribution of educational facilities and teaching staff but also accessibility, which is an important aspect of educational fairness. Accessibility refers to the ease with which students can reach educational facilities. In rural and remote areas, students face significant accessibility issues due to poor transportation and limited school resources, which directly impact their opportunities for further education. Due to transportation limitations, rural students often spend much more time and energy to reach high schools, especially those located far away. In contrast, urban students benefit from convenient transportation and a dense distribution of schools, enabling them to enjoy more accessible educational resources. The inequality in school accessibility between urban and rural areas affects students’ opportunities for further education and future development. The disparity in high school accessibility between urban and rural areas directly impacts the enrollment rates of students in these regions, exacerbating the inequality of educational opportunities. Therefore, the study analyzes the impact of high school accessibility on urban and rural high school enrollment rates within a county, revealing the role of accessibility in educational fairness. It analyzes the relationship between accessibility and urban-rural high school enrollment rates, and provides a theoretical basis for optimizing the layout of educational resources and transportation policies within the county.

Assessing transportation accessibility to schools is based on the accessibility model. Accessibility is the spatial interaction from the point of service demand to the point of service provision, and it is assessed by relying on travel mode, travel distance, and travel cost [1]. He first introduced the concept of spatial accessibility, and then more and more researchers have applied the gravity-based method [2], the two-step floating catchment area (2SFCA) method [3], and the Minimum Distance Model to measure the neighborhood accessibility to public services [4]. Methods for measuring accessibility are constantly being extended and improved to assess different types of public services such as parks [5] and schools [6]. [6] Educational facilities have also begun to be assessed and analyzed using accessibility models. Scholars studying accessibility have focused on the extent of educational service areas [7,8] spatial equity in educational services [9–12], school choice modeling for commuting [13], and accessibility analysis of junior and elementary schools [14–15]. Current research on school accessibility mainly focuses on educational facilities and school accessibility, the differences in public transportation accessibility to schools within a region, walking accessibility to schools, and other related aspects [16–18]. Current research on the distribution of urban and rural educational resources mainly focuses on differences in subject-specific educational resources, disparities in compulsory education resources between urban and rural areas, and the efficiency and equity of public education resources. There is limited research on the differences in accessibility to educational resources between urban and rural areas, and studies on the accessibility of rural and urban schools and their enrollment rates are even more scarce [19–20]. Although existing research has focused on the distribution of urban and rural educational resources, there is still a gap in empirical studies on the relationship between high school accessibility and enrollment rates.

There is a lack of research on how transportation conditions and school locations affect high school enrollment rates in urban and rural areas within a county. This study aims to fill this gap by exploring the impact of high school accessibility on the enrollment rates of urban and rural students within a county. The research helps enrich the theory of educational equity and spatial accessibility, exploring the mechanisms by which accessibility affects high school enrollment rates and providing empirical support for the theory of educational equity. The study provides a theoretical foundation and practical guidelines for the rational distribution of high school resources within the county, optimizing the transportation network, and improving educational equity. By analyzing the impact of accessibility on enrollment rates, the research could offer policy recommendations to improve educational opportunities in rural areas and increase enrollment rates, particularly through improving transportation facilities and adjusting school distribution to promote educational equity.

Based on the research questions and gaps identified above, three research hypotheses are proposed. H1: There is a positive correlation between high school accessibility and urban-rural high school enrollment rates. H2: There are significant differences in high school accessibility between urban and rural areas, and the enrollment rates of urban and rural students are affected differently by these accessibility differences. H3: The impact of high accessibility on enrollment rates is more significant in rural areas. The study will be conducted based on the verification of these research hypotheses.

The research was conducted for all senior high school in Jinzhai County. The study first investigates the spatial distribution characteristics of the 4 senior high school schools. Secondly, it uses Minimum Distance Model to investigate the minimum travel distance of the 4 high school schools to the urban and rural area in the county. Meanwhile, the research will investigate high school enrollment rate urban and rural areas. Then, the study uses descriptive and correlation analysis to investigate the relationship between urban and rural high school enrollment rates and county high school accessibility. Finally, the study uses correlation analysis to verify the research hypotheses related to urban and rural high school enrollment rates and county high school accessibility, and analyzes potential influencing factors based on the research results.

## 2. Method

### Research area

A county is one of the administrative divisions in China and is the administrative level above towns [21]. Jinzhai County is located in the western part of Anhui Province, with a land area of 3919k*m*^2^, between latitude 31°6′19″ ~ 31°48′40″ and longitude 115°22′4″ ~ 116°11′23″, with a north-south width of 77 km and an east-west length of 78 km. As of November 1, 2020, Jinzhai County had a resident population of 496,500, with 208,351 (41.96%) living in towns and 288,150 (58.04%) living in rural areas [22]. there are a total of 24 township administrative units [23]. According to the definition by the National Bureau of Statistics of China, there are two towns in the urban part of Jinzhai County, Meishan and Modern Industrial Park. The remaining 22 towns are rural areas, the names of the townships name include Mabu, Qingshan, Yanzihe, Tiantangzhai, Gubi, Wujiadian, Banzhuyuan, Tangjiahui, Nanxi, Shuanghe, Baitafan, Zhangchong, Yaofangdian, Changling, Saihuowan, Huashi, Shaha, Taoling, Geziyuan, Guanmiao, Quanjun, Tiechong [24].

It have 3 senior high schools in Jinzhai County, they are Jinzhai County No. 1 High School (JCNSHS), Nanxi Senior High School (NSHS), and Qingshan Senior High School (QSHS). The Jinzhai County No. 1 Senior High School has two campuses, the two campuses are located in different towns, so the study treats them as two separate schools. Therefore, the study will include a total of four high schools. Jinzhai County No. 1 Senior High School and Jinzhai County No. 1 Senior High School South Campus (JCNSHSSC)are located in Meishan Town and Moder Industry Park in the county’s urban construction zone. while Nanxi and Qingshan Senior High School are located in Nanxi and Qingshan Towns. All four senior high schools are open for enrollment in all townships of Jinzhai County [25]. Therefore, the study first investigates the levels of high school enrollment rates and high school accessibility in the urban and rural areas of Jinzhai County, and then examines the relationship between urban-rural high school enrollment rates and urban-rural high school accessibility.

### Data sources

The data on high school enrollment and entrance exam registration numbers primarily comes from the Jinzhai County Education Bureau and the official website of the Jinzhai County People’s Government. The data includes the number of students registered for the high school entrance exam in each town of Jinzhai County in 2023, as well as the number of students enrolled by the four high schools in Jinzhai County in 2023 [26]. The 24 towns are then classified into urban and rural areas according to the standards of the National Bureau of Statistics of China. The study will obtain the number of students registered for the high school entrance exam in urban and rural areas in 2023, as well as the number of students enrolled by the four high schools in Jinzhai County in urban and rural areas in 2023. The enrollment rates for urban and rural students in each high school are calculated by dividing the number of students enrolled in each high school by the number of students registered for the entrance exam in urban and rural areas. The original data for the study is obtained from the official website of the Jinzhai County Education Bureau, which reflects the overall trend of high school enrollment in different regions, et bland Table 1.

**Table 1:** Exam Numbers of Each Town and Enrollment Numbers of Each Senior High School.

Town Name	Exam Numbers of Each Town	Enrollment Numbers of Jinzhai No. 1 Senior High School	Enrollment Numbers of Jinzhai No. 1 Senior High School South Campus	Enrollment Numbers of Qingshan Senior High School	Enrollment Numbers of Nanxi Senior High School
Baitafan Town	361	69	28	22	39
Shuanghe Town	139	27	11	9	15
Tiantangzhai Town	166	33	13	11	18
Mabu Town	50	9	4	3	6
Liubotong Town	34	7	3	2	4
Youfangdian Town	88	17	7	6	10
Huashi Town	95	18	7	6	10
Guanmiao Town	60	11	5	4	7
Qingshan Town	94	18	7	11	5
Changling Town	89	18	6	6	10
Shahe Town	116	22	9	6	14
Taoling Town	116	22	9	7	13
Kuaishuwan Town	124	23	10	7	13
Gubei Town	327	62	24	21	36
Banzhuyuan Town	122	23	9	8	13
Tiechong Town	76	15	6	5	8
Nanxi Town	394	75	30	25	43
Guoziyuan Town	75	14	6	5	8
Wujiadian Town	236	45	18	14	57
Tangjiahui Town	319	61	24	17	37
Yanzihe Town	82	16	6	5	9
Quanjun Town	74	14	6	5	8
Modern Industry Park	587	112	45	38	64
Meishan Town	2427	465	186	156	261
Total	6251	1196	479	399	708

Reliability and Validity of the Data, Limitations of the Data. The data on the number of students registered for the high school entrance exam and high school admissions is obtained from the official website of the Jinzhai County Education Bureau. The data has been officially collected and verified. It comes from the Education Bureau’s high school entrance exam report data and the schools’ enrollment data, which ensures its high authority and reliability. The data collection and processing followed official standards and protocols, making it highly representative and accurate. However, despite the reliability of the data sources, certain regions may have incomplete statistical data due to social and cultural factors, which could slightly affect the comprehensiveness of the data.

School Locations and Transportation Network Data. The list of Senior high schools and location data for the study are primarily obtained from the official website of the Jinzhai County Education Bureau and the Jinzhai County People’s Government [27]. Jinzhai County Road Network Map, administrative boundaries of Jinzhai County, administrative divisions and latitude and longitude of administrative centers of 24 townships in Jinzhai County, latitude and longitude of 4 high schools, These data were obtained from the Amap platform [28]. With these data, the study could calculate the accessibility of urban and rural areas to each senior high school and further analyze the factors affecting accessibility. The accessibility indicator is the shortest travel distance, which could be calculated using GIS technology. These indicators measure the accessibility level for students traveling from different towns to each high school. The location data for the senior high schools includes the geographical coordinates of the four high schools in Jinzhai County, as well as the specific locations of each school within the county. The data for the main transportation network in Jinzhai County, including city roads, rural roads, and major transportation routes, is sourced from Amap [29].

Reliability and Validity of the Data, Limitations of the Data. The data comes from government departments and online mapping platforms, making it highly reliable and valid. The transportation network data provided by the online mapping platform and the school location data provided by the Jinzhai County Education Bureau are publicly available data that have been verified by authoritative sources. Additionally, the use of GIS technology enhances the precision of spatial analysis, allowing for accurate calculation of the accessibility from each region to the schools, ensuring the accuracy of the research results. Although the data sources are authoritative, there are still certain limitations. The shortest travel distance method used in the study may overlook certain real-world factors, such as road congestion or traffic accidents, which can affect the actual travel time for students. As a result, there may be some error in the data analysis.

After data collection is completed, the study performs checks and cleaning on all raw data to ensure its accuracy and consistency. Outliers are handled by either supplementation or removal. The high school enrollment data is checked for duplicate or erroneous records, with inconsistent data being deleted or corrected. The transportation network data is verified to ensure that all road information is accurate, and any potential errors are corrected.

### Analysis methods

Accessibility is the spatial interaction from the point of service demand to the point of service provision [30]. The accessibility of each senior high school in urban and rural areas can be evaluated using the shortest travel distance method, which calculates the shortest path from a given location to the target location in order to obtain the urban and rural accessibility scores for each high school [31]. In this study, the OD cost matrix method from GIS network analysis tools is used to calculate the shortest travel distances from the four high schools in Jinzhai County to the 24 towns, obtaining the accessibility scores for each of the four high schools to the 24 towns. Due to the lack of data on students’ home locations in towns and villages, the study uses the government seat of each town as the endpoint. The study sets the four high schools as starting points and the government seat of each town as the endpoint. Through GIS, the shortest distance from each high school to the 24 towns is calculated and then divided by the total number of towns in urban or rural areas, thus calculating the average accessibility score of each high school to the urban or rural regions. The specific formula for the study is as follows:


$$C_{i}=\frac{1}{N}\sum_{j=1}^{n}D_{ij}$$

$C_{i}$ represents the average travel distance (average accessibility score) for high school i to the urban or rural area. $D_{ij}\;$indicates the shortest travel distance (accessibility score) from high school i to urban or rural town j. N is the total number of towns in the urban or rural area.

Normalization of Accessibility Scores. Since accessibility scores are based on distance, such as thousands or tens of thousands, these values may differ significantly from enrollment rates in the analysis. Therefore, the study normalizes the data. Given that the urban area has only 2 towns, which is a very small sample size, the study first groups the urban and rural areas separately, then normalizes the data, calculating the minimum, maximum, mean, and standard deviation for each group individually. This approach helps to avoid bias caused by the small sample size in the urban area. The normalization formula is as follows:


$$C_{i}^{\prime }=\frac{C_{i}-C_{\min}}{C_{\max}-C_{\min}}$$

$C_{i}^{\prime }$ represents the normalized accessibility score for high school i in the urban or rural area. $C_{\min}$ is the minimum accessibility score in the urban or rural area, and $C_{\max}$ is the maximum accessibility score in the urban or rural area.

Senior High school enrollment rate is the proportion of students in a region who enter high school. This study uses the enrollment data of the four high schools in Jinzhai County and the number of students registered for the high school entrance exam in urban and rural areas to calculate the enrollment rate for each high school in both urban and rural areas. The specific calculation formula is as follows:


$$Enrollment\;rate=\frac{High\;School\;Enrollment\;Numbers}{high\;school\;entrance\;exam\;registration\;numbers.}*100\;\%$$

After calculating the urban and rural high school enrollment rates, this study will use descriptive statistics and correlation analysis to explore the differences in enrollment rates between urban and rural areas and their underlying causes. The analysis will focus on how differences in high school accessibility affect urban and rural enrollment rates, and further examine the relationship between enrollment rates and accessibility. By analyzing these differences, the study aims to reveal the spatial distribution of high school enrollment opportunities and provide theoretical support for optimizing the allocation of educational resources within the county.

The study uses descriptive analysis to reveal the basic characteristics and distribution of enrollment rates and accessibility. It first uses descriptive analysis to provide a preliminary summary and overview of the high school enrollment rates and accessibility data in Jinzhai County, identifying the differences in enrollment rates and school accessibility between urban and rural areas. The study summarizes urban and rural enrollment rates and high school accessibility using measures such as mean, standard deviation, maximum, and minimum values.

The study uses non-parametric tests to analyze the differences in enrollment rates and accessibility between urban and rural areas. The Mann-Whitney U test is a non-parametric method suitable for small sample sizes (this study has a sample size of 4) and non-normally distributed data [32]. The test is based on the ranks of the data, and by calculating the rank sums $R_{1}$ and $R_{2}$ for the two samples, the null hypothesis assumes that the medians of the two samples are the same, while the alternative hypothesis suggests that the medians of the two samples are significantly different. The significance level is set at *p* < 0.05. Through the Mann-Whitney U test, this study aims to evaluate whether there are statistically significant differences in urban and rural accessibility and enrollment rates, and provide a basis for further exploring urban-rural educational equity. The formula is as follows:


$$U=n_{1}n_{2}+\frac{n_{1}(n_{1}+1)}{2}-R_{1}$$

U is the test statistic. $n_{1}$ is the sample size of the first group, $n_{2}$ is the sample size of the second group, and $R_{1}$ is the rank sum of the first group (i.e., the total sum of the ranks of the first group after combining the two datasets).

The research uses Kendall’s tau-b correlation coefficient to explore the relationship between senior high school enrollment rates and high school accessibility in urban and rural areas. The Kendall’s tau-b correlation coefficient is suitable for small samples and non-normally distributed data. Since there are only four senior high schools in Jinzhai County, the sample size is small, making the Kendall’s tau-b correlation coefficient an appropriate method to test whether the hypotheses hold. Its formula is as follows:


$$\tau_{b}=\frac{C-D}{\sqrt{\left(C+D+T_{x})(C+D+T_{y}\right)}}$$

C is the number of concordant pairs, D is the number of discordant pairs, $T_{x}$ is the number of tied values in variable X, and $T_{y}$ is the number of tied values in variable Y. The value of Kendall’s tau-b correlation coefficient ranges from −1 to +1, where +1 indicates a perfect positive correlation, meaning the rankings of the two variables are completely aligned. −1 indicates a perfect negative correlation, meaning the rankings of the two variables are completely opposite. 0 indicates no correlation, meaning the rankings of the two variables are unrelated.

In the study, the Kendall’s tau-b correlation coefficient is used to perform the correlation analysis. The study will examine whether there is a significant positive or negative correlation between high school accessibility and urban-rural high school enrollment rates. This analysis will help understand the extent to which accessibility impacts enrollment rates and provide a foundation for subsequent analysis.

## 3. Results

### Admission rate data

**Fig 1:**
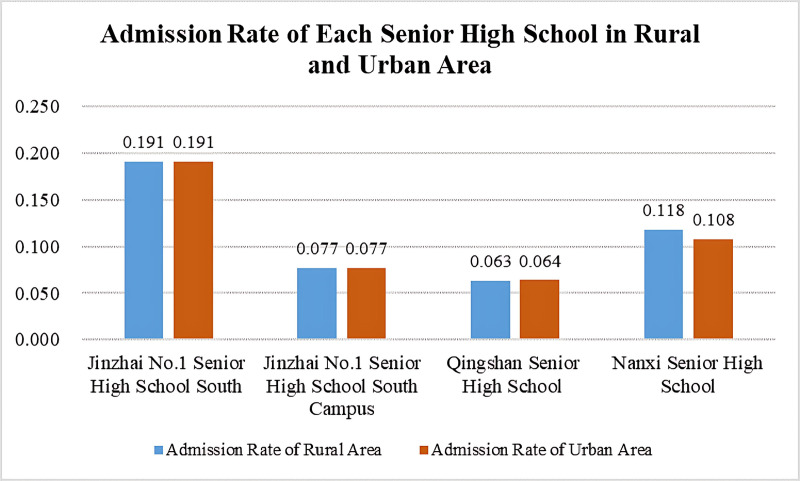
Admission Rate of Each Senior High School in Rural and Urban Area.

The sample for this study includes the enrollment rates of the four high schools in Jinzhai County in urban and rural areas, with 2 towns in the urban area and 22 towns in the rural area. The enrollment rates for each high school in the urban and rural areas were obtained using the above formula, as shown in Fig 1. The results show slight differences in the enrollment rates of each high school between the urban and rural areas. To verify the above research hypotheses, the study will further analyze the data.

**Fig 2:**
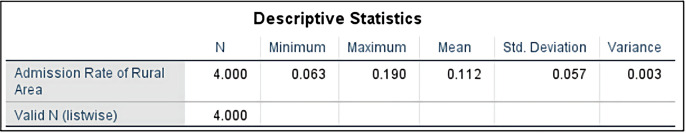
Descriptive Statistical Results of Rural Admission Rate.

**Fig 3:**
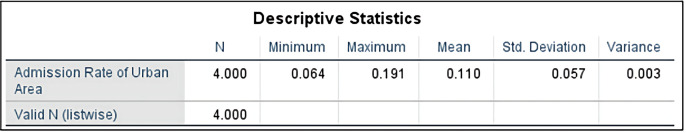
Descriptive Statistical Results of Urban Admission Rate.

The study performed descriptive statistical analysis on the high school enrollment rates in rural and urban areas of Jinzhai County. The results show that the enrollment rates of the four high schools in the rural areas of Jinzhai County range from 6.3% to 19.0%, with an average enrollment rate of 11.2%, as shown in Fig 2. The standard deviation of the enrollment rates for each high school is 5.7%, and the variance is 0.003. In the urban areas, the enrollment rates of the four high schools range from 6.4% to 19.1%, with an average enrollment rate of 11.0%, as shown in Fig 3. The standard deviation of the enrollment rates for each high school is 5.7%, and the variance is 0.003. These results indicate that there is a certain degree of difference in the enrollment rates between rural and urban areas for the four high schools in Jinzhai County.

**Fig 4:**
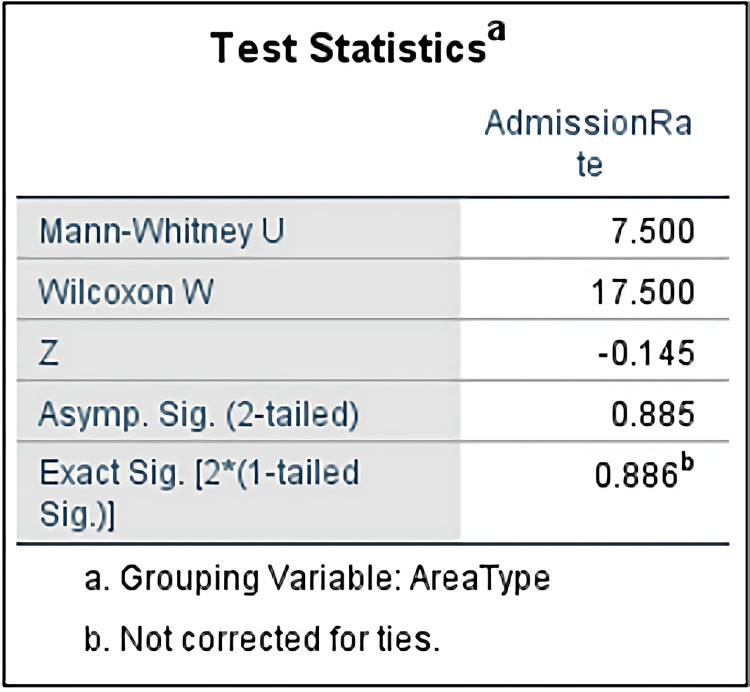
Mann-Whitney U Test Statistics Results of Rural and Urban Admission Rate.

To verify whether the difference in high school enrollment rates between urban and rural areas reaches a statistically significant level, the study uses the Mann-Whitney U test to analyze the differences in enrollment rates between urban and rural areas, as shown in Fig 4. The test results show that the Mann-Whitney U value is 7.500, the Wilcoxon W value is 17.500, and the standardized statistic Z value is −0.145. The asymptotic significance level (Asymp. Sig. [2-tailed]) is 0.885, and the exact significance level (Exact Sig. [2*(1-tailed Sig.)]) is 0.886, both of which are much larger than the commonly used significance threshold (p > 0.05). Therefore, the statistical results indicate that the difference in admission rates between urban and rural areas in Jinzhai County does not reach a statistically significant level.

The study found that the high school enrollment rates in urban and rural areas of Jinzhai County did not reach a statistically significant level. By using the Mann-Whitney U test to compare the high school enrollment rates in urban and rural areas for the four high schools in Jinzhai County, the results indicate that there is no significant difference between the two. The conclusion reveals that, statistically, the high school enrollment rates in urban and rural areas of this region are equal, despite the typical differences in educational resources, economic conditions, and cultural backgrounds between urban and rural areas.

The difference in high school enrollment rates between urban and rural areas in Jinzhai County is small and is influenced by several factors such as enrollment policies, educational resources, and accessibility. First, the formulation and implementation of enrollment policies may have contributed to promoting educational equity between urban and rural areas. For example, high school enrollment quotas are allocated based on the number of students registered for the high school entrance exam in each middle school, which results in similar enrollment rates for each high school in urban and rural areas. Secondly, the distribution of educational resources may be relatively balanced. In recent years, the Jinzhai County government has increased investments in rural education, leading to significant improvements in infrastructure and teaching resources, thereby narrowing the education gap between urban and rural areas. Additionally, the Jinzhai County government may have implemented policies and measures to ensure that students in both urban and rural areas have equal opportunities to compete for high school admissions. Finally, while the accessibility score for some areas in Jinzhai County to attend high school is relatively low, students are willing to travel to farther schools in order to attend their target high schools.

### Accessibility data

**Fig 5:**
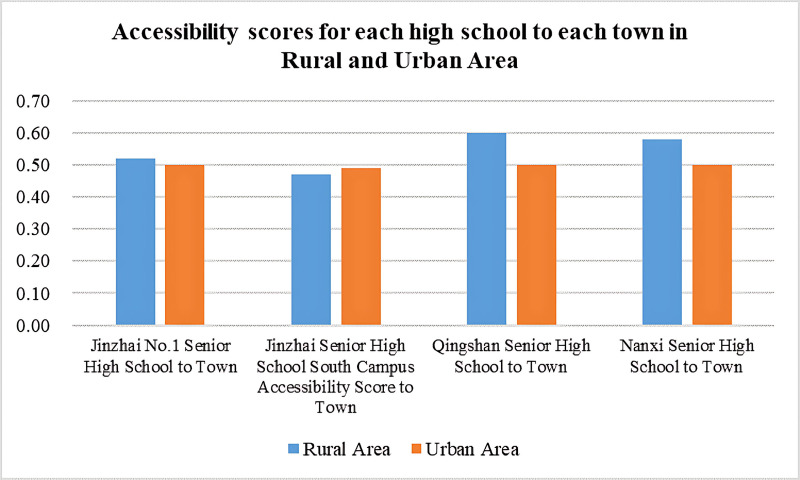
Accessibility scores for each high school to each town in Rural and Urban Area.

The sample for this study includes four high schools in Jinzhai County and 24 towns, with 2 towns in the urban area and 22 towns in the rural area. Using GIS tools and the above formulas. the accessibility scores for each high school in both urban and rural areas were obtained, as shown in Fig 5. Fig 5 presents the normalized accessibility scores for the four high schools. The results show slight differences in the accessibility scores of each high school between the urban and rural areas. To verify the above research hypotheses, the study will further analyze the data.

**Fig 6:**
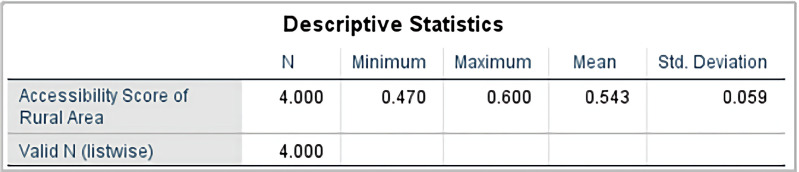
Descriptive Statistical Results of Accessibility of Rural Area.

**Fig 7:**
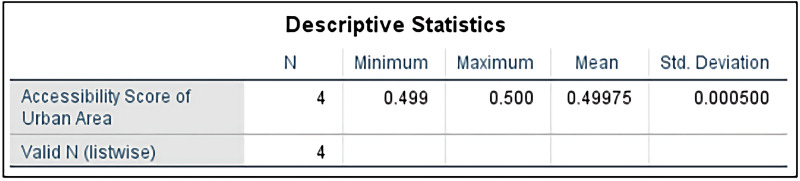
Descriptive Statistical Results of Accessibility of Urban Area.

The study performed descriptive analysis on the high school accessibility in rural and urban areas of Jinzhai County. The results show that the accessibility scores of the four high schools in Jinzhai County in the rural areas range from 0.47 to 0.6, with an average accessibility score of 0.543, as shown in Fig 6. The standard deviation of the accessibility scores for each high school in both urban and rural areas is 0.059. In the urban areas, the accessibility scores of the four high schools range from 0.499 to 0.5, with an average accessibility score of 0.49975, as shown in Fig 7. The standard deviation of the accessibility scores for each high school is 0.0005. This indicates that there is a certain degree of difference in the accessibility of the four high schools in Jinzhai County between the rural and urban areas.

**Fig 8:**
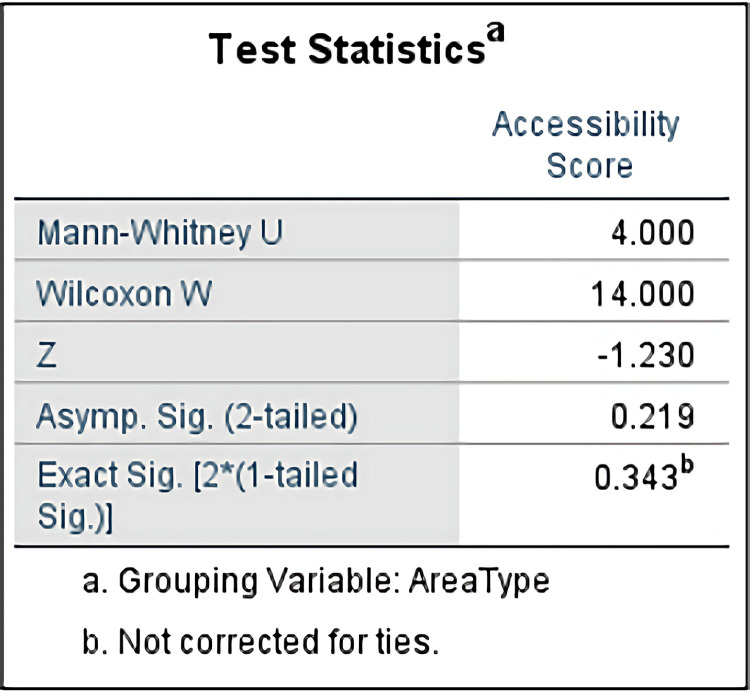
Mann-Whitney U Test Statistics Results of Rural and Urban Admission Rate.

To verify whether the difference in accessibility between urban and rural areas reaches a statistically significant level, this study uses the Mann-Whitney U test to compare the Accessibility Scores of urban and rural high schools, with the grouping variable being Area Type, as shown in Fig 8. The test results show that the Mann-Whitney U value is 4.000, the Wilcoxon W value is 14.000, and the standardized statistic Z value is −1.230. The asymptotic significance level is 0.219, and the exact significance level is 0.343, both of which are greater than the commonly used significance threshold (p > 0.05). Therefore, the statistical results indicate that the difference in Accessibility Scores between urban and rural high schools does not reach a statistically significant level.

This study compared the accessibility scores of urban and rural areas for the four senior high schools in Jinzhai County using the Mann-Whitney U test. The results show that there is no significant difference between the two. In Jinzhai County, there is no noticeable gap in high school accessibility between urban and rural areas, meaning that students in both urban and rural areas have equal accessibility to high schools.

The lack of a significant difference in the urban and rural accessibility scores of the four high schools in Jinzhai County may be closely related to factors such as the school’s location and transportation infrastructure. First, the distribution of educational resources and the location of schools may have contributed to the balance in accessibility scores. For example, two of Jinzhai County’s high schools are located in rural areas, and two are located in urban areas, which makes the school placement in both urban and rural areas relatively reasonable, minimizing the distance gap and ensuring fairness in high school accessibility for students in both areas. Furthermore, Jinzhai County has made balanced progress in transportation infrastructure development between urban and rural areas. In recent years, the national and local governments have invested substantial resources into improving transportation conditions in rural areas, such as building rural roads, which has made the travel distances for urban and rural students to reach high schools similar. This result provides valuable insights for further exploring urban-rural educational equity and resource balance, indicating that Jinzhai County has achieved a certain level of balance in educational accessibility between urban and rural areas.

### Accessibility and enrollment rate analysis

**Fig 9:**
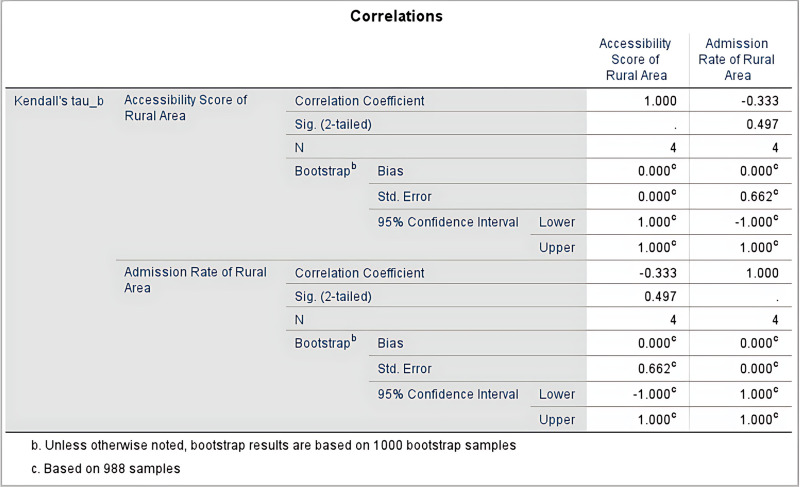
Correlations Analyze Results of Admission Rate and Accessibility of Rural Area.

The study used Kendall’s tau-b correlation coefficient to analyze the correlation between accessibility scores and enrollment rates in rural areas, testing the related research hypothesis, as shown in Fig 9. The results indicate that the correlation coefficient is −0.333, suggesting a moderate negative correlation, meaning that as accessibility increases in rural areas, the enrollment rate may show a declining trend. However, the two-tailed significance level (Sig. [2-tailed]) is 0.497, which is greater than the commonly used significance threshold (p > 0.05), indicating that the correlation is not statistically significant. Bootstrap analysis, based on 988 resamples, shows a bias of 0.000, a standard error of 0.662, and a 95% confidence interval of [−1.000, 1.000], further supporting the conclusion that the correlation is not significant. The small sample size of this study (N = 4) may affect the stability and reliability of the results.

Although the variation in high school accessibility scores in rural areas is large, the improvements in transportation infrastructure and the government’s support for rural education in recent years have significantly enhanced the school attendance conditions for rural students. Therefore, despite lower accessibility, the enrollment opportunities for rural students have not been significantly affected, as two of the high schools are located in rural areas. The government’s educational policies, especially the allocation of enrollment quotas based on the number of students registered for the entrance exam in each middle school, have helped rural students overcome transportation barriers and ensured that they can successfully enter high school.

**Fig 10:**
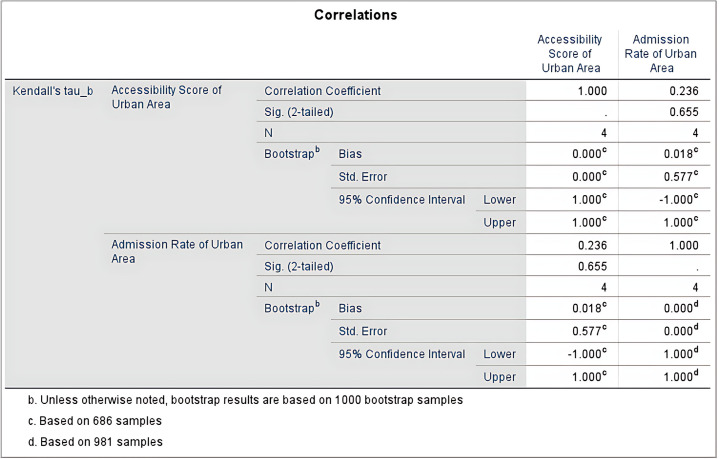
Correlations Analyze Results of Admission Rate and Accessibility of Urban Area.

The study used Kendall’s tau-b correlation coefficient to analyze the correlation between accessibility scores and high school enrollment rates in urban areas, as shown in Fig 10. The results show that the correlation coefficient is 0.236, indicating a moderate correlation, meaning that as accessibility increases in urban areas, enrollment rates may show an upward trend. However, the two-tailed significance level (Sig. [2-tailed]) is 0.655, which is greater than the commonly used significance threshold (p > 0.05), indicating that the correlation is not statistically significant. Bootstrap analysis, based on 988 resamples, shows a bias of 0.018, a standard error of 0.577, and a 95% confidence interval of [−1.000, 1.000], further supporting the conclusion that the correlation is not significant. The small sample size of this study (N = 4) may affect the stability and reliability of the results.

In urban areas, there is no significant difference between high school enrollment rates and high school accessibility, and the accessibility scores of high schools in urban areas are more evenly distributed. This may be due to the well-developed transportation infrastructure in urban areas, which makes it easier for students to access any high school. Additionally, since high school enrollment quotas in Jinzhai County are allocated based on the number of students registered for the high school entrance exam, accessibility scores have not had a significant impact on the enrollment rates.

In summary, there is no significant difference between urban and rural high school accessibility and enrollment rates, primarily due to improvements in transportation infrastructure and effective support from government enrollment policies. Although urban areas have higher accessibility, the enrollment rates were not significantly affected by the increase in accessibility due to the allocation of enrollment quotas based on the number of students registered for the entrance exam. Despite the large variations in high school accessibility in rural areas, policy support and improved transportation conditions have successfully bridged the gap between urban and rural areas, ensuring equal enrollment opportunities for rural students. As a result, the differences in high school enrollment rates and accessibility between urban and rural areas in Jinzhai County have gradually narrowed, reflecting urban-rural educational equity.

This study did not find a significant positive correlation between high school accessibility and high school enrollment rates in urban and rural areas. Using Kendall’s tau-b correlation coefficient, the study performed correlation tests on the high school accessibility and enrollment rates in both urban and rural areas. The correlation between urban high school accessibility and enrollment rates was not significant (correlation coefficient of 0.236, p-value of 0.655). Similarly, there was no significant negative correlation between high school accessibility and enrollment rates in rural areas (correlation coefficient of −0.333, p-value of 0.497). Therefore, the research hypothesis “There is a positive correlation between high school accessibility and urban-rural high school enrollment rates” is not supported.

Since the differences in accessibility are not significant, the enrollment rates of students in urban and rural areas are not notably influenced by accessibility differences. According to the research results, there is no significant gap in high school accessibility between urban and rural areas. As the differences in accessibility are not significant, the impact of accessibility on enrollment rates in urban and rural areas is also not noticeable. Therefore, the difference in high school accessibility between urban and rural areas is small, and it does not show a significant effect on enrollment rates. The research hypothesis “There is a large difference in high school accessibility between urban and rural areas, and the enrollment rates of urban and rural students are differently affected by accessibility differences” is not supported.

The impact of high school accessibility on enrollment rates in rural areas was not as significant as expected. In this study, there was no significant negative correlation between high school enrollment rates and accessibility in rural areas. The bootstrap analysis also did not show a significant correlation (bias of 0.000, standard error of 0.662, 95% confidence interval of [−1.000, 1.000]). Therefore, the research hypothesis “The impact of high accessibility on enrollment rates is more significant in rural areas” is not supported.

This study did not validate all of the research hypotheses. Although Hypotheses H1, H2, and H3 proposed a correlation between high school accessibility and enrollment rates, the empirical analysis found that the correlation between high school accessibility and enrollment rates in both urban and rural areas was not significant, and the differences in high school accessibility between urban and rural areas did not show a significant impact. This may be due to the gradual balancing of educational resources, improvements in transportation infrastructure, and government policy support in Jinzhai County, which has led to a gradual narrowing of the differences in educational accessibility and enrollment rates between urban and rural areas. Therefore, none of the three hypotheses in this study were supported.

## 4. Discussion

In both urban and rural areas of Jinzhai County, despite the existence of some differences in accessibility, there was no significant correlation between high school enrollment rates and high school accessibility. In urban areas, despite higher high school accessibility, there was no significant positive correlation with enrollment rates; in rural areas, despite relatively lower accessibility, there was no significant negative correlation either. Overall, the differences in high school accessibility and enrollment rates between urban and rural areas were small and did not show significant effects in statistical analysis.

The existing research results did not support the hypothesized positive correlation, which may be influenced by several factors. First, in recent years, the government’s balanced investment in urban and rural educational resources and improvements in infrastructure, especially in terms of school locations and rural infrastructure, have effectively reduced the gap in accessibility between urban and rural areas. Through enrollment policies and educational resource support, the conditions for rural students to attend school have significantly improved, reducing the impact of accessibility on enrollment rates. Second, in urban areas, educational resources are relatively saturated. Although high school accessibility is higher, intense competition for enrollment and limited slots may have suppressed the effect of accessibility on enrollment rates. Additionally, socio-economic background and family income may play a more crucial role in educational opportunities in certain areas.

To further achieve urban-rural educational equity, the government should optimize enrollment policies, increase investment in rural education, and expand rural road construction. For urban areas, the government can consider adjusting high school enrollment policies or increasing priority slots for rural students, addressing the issue of over-concentration of educational resources in areas with high accessibility, and ensuring that more students have equal educational opportunities. While the accessibility of education in rural areas has improved, the government should continue to strengthen infrastructure development, especially in remote areas, to ensure further balanced distribution of transportation and educational resources. Through these policy supports, it is possible to effectively promote the balanced development of urban and rural education, narrow the gap in educational resources and opportunities, and ensure that more students have equal opportunities for further education.

The study’s findings indicate that there is no significant correlation between high school accessibility and enrollment rates in urban and rural areas of Jinzhai County. This result highlights the ongoing trend of the gradual balancing of urban and rural educational resources, especially the efforts made by the government in improving educational accessibility in rural areas and providing policy support. Although urban areas have higher accessibility, the enrollment rates have not been significantly affected due to the saturation of educational resources. In rural areas, improvements in enrollment policies and infrastructure have effectively narrowed the gap in high school enrollment rates between urban and rural areas, ensuring equal educational opportunities. This finding offers a new perspective on existing theories, showing that the relationship between educational accessibility and enrollment rates is not only influenced by transportation conditions but also closely related to government policies, socio-economic background, and other factors.

The study reveals the relationship between urban-rural educational accessibility and enrollment rates, particularly in the context of policy support and resource balance, challenging the traditional assumption that higher accessibility inevitably leads to higher enrollment rates. Future research could further explore similar situations in other regions, analyzing the interaction between educational accessibility and enrollment rates under different policy contexts. Particularly with larger sample sizes and longer time spans, it may uncover more valuable conclusions about educational equity and resource allocation.

## 5. Conclusion

The main goal of this study is to explore the impact of high school accessibility on urban and rural students’ enrollment rates within a county and to analyze the mechanisms through which the differences in urban and rural high school accessibility affect enrollment rates. The study aims to fill the gap in existing empirical research, especially focusing on how transportation conditions and school distribution affect urban and rural high school enrollment rates within a county. The research questions focus on whether there is a significant relationship between high school accessibility and enrollment rates in urban and rural areas, whether the differences in high school accessibility between urban and rural areas have a significant impact on enrollment rates, and whether high accessibility has a more significant effect on rural enrollment rates.

The main findings of this study can be summarized in two parts. First, no significant positive correlation was found between high school accessibility and enrollment rates in urban and rural areas. Although urban areas have higher high school accessibility, the enrollment rates were not significantly affected due to the saturation of educational resources. In rural areas, despite lower high school accessibility, improvements in transportation policies and infrastructure ensured that enrollment rates did not significantly decline. Second, in terms of differences in high school accessibility between urban and rural areas, no significant differences were found. Urban and rural students did not exhibit significant gaps in accessibility, meaning that accessibility had no clear impact on enrollment rates in an urban-rural context. This finding indicates that Jinzhai County has achieved a certain degree of balance in the allocation of urban and rural educational resources.

The contributions of this study include theoretical contributions, practical contributions, and a methodological framework. The study provides a new perspective for understanding urban-rural educational disparities, particularly in the relationship between accessibility and enrollment rates. By revealing the mechanisms through which accessibility affects urban and rural enrollment rates, this research deepens the understanding of the interrelationships between educational equity, spatial accessibility, and opportunities for further education. This study offers valuable insights for policymakers, especially in optimizing the distribution of urban and rural educational resources, improving transportation networks, and promoting educational equity. The study shows that despite differences in accessibility between urban and rural areas, educational equity can be effectively promoted through policy support and infrastructure improvement. Additionally, the study provides a methodological framework for evaluating the relationship between urban-rural high school accessibility and enrollment rates. This framework offers a systematic analytical tool for future related research and provides theoretical support and practical analysis methods for policymakers in optimizing the allocation of educational resources.

The main limitations of this study lie in the small sample size and the fact that other external factors were not considered. The study is limited to data from Jinzhai County, which may affect the external validity and generalizability of the results. Moreover, the study used descriptive and correlation analysis methods, which, while revealing the relationship between accessibility and enrollment rates, cannot establish a causal relationship. The study hypothesized that the differences between urban and rural areas are primarily reflected in accessibility, but other factors, such as policy, cultural background, and family economic conditions, may also influence enrollment rates, and these factors may not have been fully considered.

Suggestions for Future Research. Based on the limitations and unanswered questions, future research could expand the sample size to include more urban and rural areas to improve the representativeness of the results. Experimental designs or longitudinal research methods could be used to explore the causal relationship between accessibility and enrollment rates. Furthermore, future studies could incorporate additional control variables (such as family background, policy factors, etc.) to further analyze their impact on enrollment rates, and consider a multidimensional analytical framework to explore the effects of family background, socio-economic status, and educational policies on enrollment rates and accessibility. Finally, cross-regional comparative studies should also be considered. Future research could compare the educational systems of different countries or regions to explore the relationship between educational opportunities and enrollment rates in different contexts.

This study provides valuable insights for education policymakers by analyzing the relationship between urban and rural accessibility and enrollment rates, especially in promoting educational equity and resource balance. Despite some limitations, the study contributes to providing theoretical support and practical guidance for addressing urban-rural educational inequality, particularly by offering targeted policy recommendations to improve educational opportunities and enrollment rates in rural areas. Future research will further broaden the scope of this field, continuing to explore the profound impact of educational equity and spatial accessibility on students’ opportunities for further education.
